# Low ecological representation in the protected area network of China

**DOI:** 10.1002/ece3.4175

**Published:** 2018-05-24

**Authors:** Haigen Xu, Mingchang Cao, Zhi Wang, Yi Wu, Yun Cao, Jun Wu, Zhifang Le, Peng Cui, Hui Ding, Wanggu Xu, Hua Peng, Jianping Jiang, Yuhu Wu, Xuelong Jiang, Zhiyun Zhang, Dingqi Rao, Jianqiang Li, Fumin Lei, Nianhe Xia, Lianxian Han, Wei Cao, Jiayu Wu, Xin Xia, Yimin Li

**Affiliations:** ^1^ Nanjing Institute of Environmental Sciences Ministry of Environmental Protection Nanjing China; ^2^ College of Forest Resources and Environment Nanjing Forestry University Nanjing China; ^3^ College of Life Sciences Nanjing University Nanjing China; ^4^ Kunming Institute of Botany Chinese Academy of Sciences Kunming China; ^5^ Chengdu Institute of Biology Chinese Academy of Sciences Chengdu China; ^6^ Northwest Institute of Plateau Biology Chinese Academy of Sciences Xining China; ^7^ Kunming Institute of Zoology Chinese Academy of Sciences Kunming China; ^8^ Institute of Botany Chinese Academy of Sciences Beijing China; ^9^ Wuhan Botanical Garden Chinese Academy of Sciences Wuhan China; ^10^ Institute of Zoology Chinese Academy of Sciences Beijing China; ^11^ South China Botanical Garden Chinese Academy of Sciences Guangzhou China; ^12^ College of Forestry Southwest Forestry University Kunming China; ^13^ Institute of Applied Ecology Chinese Academy of Sciences Shenyang China; ^14^ College of Urban and Environmental Sciences Peking University Beijing China; ^15^ Faculty of Science Jiangsu University Zhenjiang China

**Keywords:** biodiversity, complementarity, conservation gaps, species, threatened species

## Abstract

Protected areas are considered as an essential strategy to halt the decline of biodiversity. Ecological representation in protected areas is crucial for assessment on the progress toward conservation targets. Although China has established a large number of protected areas since the 1950s, ecological representation of protected areas is poorly understood. Here, we performed the complementarity analysis to evaluate ecological representation of protected areas in China. We used a database of the geographical distribution for 10,396 woody plant species, 2,305 fern species, 406 amphibian species, 460 reptile species, 1,364 bird species, and 590 mammal species from 2,376 counties across China. We identified complementary sets of counties for all species or threatened species of plant and vertebrate species using a complementarity algorithm. We evaluated ecological representation of 3,627 protected areas and discerned conservation gaps by comparing the distribution of protected areas with complementary sets. The results show that the spatially representative and complementary sites for biodiversity are poorly covered, and a fairly large proportion of protected areas is not designed to efficiently represent biodiversity at the national scale. Our methodology can serve as a generic framework for assessment on ecological representation of protected areas at the national scale.

## 1 INTRODUCTION

Biodiversity has continued to decline over the past four decades (Butchart et al., 2010). After a failure to achieve a significant reduction in the rate of biodiversity loss by 2010, parties to the Convention on Biological Diversity (CBD) adopted the Strategic Plan for Biodiversity 2011–2020 and the Aichi Targets (Butchart et al., 2010; CBD, 2010). Aichi Target 11 was set to conserve at least 17% of terrestrial and inland water areas, and 10% of coastal and marine areas by 2020, through ecologically representative systems of protected areas (PAs) (Convention on Biological Diversity (CBD), 2010). As an essential strategy for biodiversity conservation, PAs’ establishment can facilitate the achievement of the global targets (Butchart et al., 2012; Juffe‐Bignoli et al., 2014). Furthermore, assessment on ecological representation of PAs offers guidance to efficient designation of land, financial, and human resources for in situ biodiversity conservation. Ecoregions are adopted as a useful proxy to evaluate ecological representativeness at the global scale (Juffe‐Bignoli et al., 2014; Pimm et al., 2014). However, it is too coarse to apply at the national level (Juffe‐Bignoli et al., 2014), and not efficient to cover species diversity (Venter et al., 2014). Thus, more accurate approaches based on species’ geographical distribution are urgently needed for assessing ecological representation of PAs at the national scale.

China is considered as one of the “megadiversity” countries in the world (Brooks et al., 2006; Liu et al., 2003). It harbors over 10% of the total number of plant and vertebrate species worldwide (Liu et al., 2003; Tang, Wang, Zheng, & Fang, 2006). As the fastest developing country in the world, China is facing historically unprecedented pressures from the largest population and rapid economic growth (Liu & Diamond, 2005; Liu et al., 2003; World Bank, 2015). It poses great threats to biodiversity and creates barrier to the ongoing conservation. Since the 1950s, China has established a large number of PAs for in situ biodiversity conservation (Wu et al., 2011; Zhang, Luo, Mallon, Li, & Jiang, 2016). Currently, China has almost realized the Aichi Target 11 in terms of PA coverage (approximately 16.8% as compared with 17% of the target, see section 2). Furthermore, the ecological representation of PAs in China requires more systematic evaluation. Recent studies evaluated the ecological representation of PAs (e.g. nature reserves) across China in terms of terrestrial ecoregions, biodiversity priority areas, and vegetation types (Wu et al., 2011; Zhang et al., 2016). However, species diversity has not been considered in assessing the ecological representation of PAs. Species diversity is the important elements in ecological representation.

In this study, we provided a species‐based approach to evaluate the ecological representation of PAs across China. First, we identified complementary sets (CSs) of counties where all species or threatened species are covered for biodiversity conservation, using a complementarity algorithm (Colwell & Coddington, 1994). Then, we evaluated the ecological representation of PA network and identified conservation gaps by comparing PAs with CSs across China. Finally, we presented proposals for improving ecological representation of PAs across China.

## 2 METHODS

### 2.1 Species data

We constructed a database of the geographical distribution for 10,396 woody plant species, 2,305 fern species, 406 amphibian species, 460 reptile species, 1,364 bird species, and 590 mammal species from 2,376 counties across China (Xu, Cao, Wu, & Ding, 2013; Xu et al., 2015, 2016). The checklist of species was obtained from the Catalogue of Life China 2011 Annual Checklist (The Biodiversity Committee of Chinese Academy of Sciences, 2011) and Red Data Book of Biodiversity (Ministry of Environmental Protection of China and Chinese Academy of Sciences, 2013, 2015). This database was compiled based on presence records from (a) approximately 900 literatures on the distribution of vertebrates and plants from 1970 to 2012, (b) collection information of specimens in herbaria of more than 20 institutes and universities, and (c) ground observation information of such taxa based on records of field surveys during 2000 and 2010 by experts from more than 11 institutes of Chinese Academy of Sciences and over 14 universities (Xu et al., 2015, 2016). To improve the data quality, we organized more than 20 expert meetings and invited over 100 experts specialized in a variety of specific taxa to check the data on spatial distribution of each species across China based on a GIS information system that we developed for species distribution at the county level. Species in marine ecosystems, cultivated or bred species in botanical gardens, zoos or farms, and exotic species were eliminated from this study. To our current knowledge, this database covers nearly all species of the six taxa native to China (>98%) and is the most comprehensive database ever developed in the country. We mainly used “county” as the basic planning unit in this study (Xu et al., 2015, 2016). Moreover, such units were also considered as an assessment unit, respectively, that is, the urban area of a municipality, the urban area of a capital city in a province or autonomous region, the urban area of a city at prefectural level, and a special administrative region (e.g. Hong Kong, Macao). In total, 2,376 assessment units (henceforth “counties”) were included in this study (Xu et al., 2015, 2016).

Threatened species are those species that are critically endangered, endangered, or vulnerable, as defined by IUCN Red List Categories and Criteria (Version 3.1). In the dataset, 1,490 woody plant species, 148 fern species, 176 amphibian species, 138 reptile species, 146 bird species, and 156 mammal species have been listed as threatened according to China’s Red List (Ministry of Environmental Protection of China and Chinese Academy of Sciences, 2013, 2015).

### 2.2 Protected areas

We primarily focused on PAs in terrestrial and inland water areas that are crucial for the achievement of Aichi Target 11. In this study, we made an assessment of 3,627 PAs which cover a total area of 161.7 million ha and account for approximately 16.8% of the terrestrial territory of China. Nature reserves of geologic relicts and paleontologic relicts and marine nature reserves were not considered in this study as they are basically irrelevant to terrestrial biodiversity (Xu et al., 2008). Nature reserves that are only depicted on paper and lack valid information on geographical location were also excluded. A total of 199 nature reserves eliminated in this study cover a total area of 3.61 million ha accounting for <0.4% of the country’s land area. Thus, PAs considered in our study represent the majority of PAs in China.

We collected data on the name, area, type, location or distribution boundary, presence and area in counties, and year of establishment of PAs from 1993 to 2013. Data on nature reserves in mainland China were derived from the Ministry of Environmental Protection (http://www.mep.gov.cn/). Data on national parks in mainland China were obtained from the Ministry of Housing, Urban‐rural Development (http://www.mohurd.gov.cn/). Data on national forest parks in mainland China were from the State Forestry Administration (http://www.forestry.gov.cn/). Data on PAs in Taiwan, Hong Kong, and Macao respectively were derived from the websites of their relevant administrative bodies. If a PA is intersected with several counties and data on its area in each county were unavailable, we allocated the area of the PA evenly to each county. PAs were recorded either as polygons and/or as points.

### 2.3 Complementarity analysis

Biodiversity is not congruent across taxa (Orme et al., 2005; van Jaarsveld et al., 1998) and unevenly distributed around the world. A PA network should provide adequate coverage of all components of biodiversity. According to systematic conservation planning (Margules & Pressey, 2000; Pressey, Humphries, Margules, Vane‐Wright, & Williams, 1993), the overall effectiveness of PA network depends not only on their species richness but also on how well they complement one another biologically (Pressey et al., 1993). Reserve selection methodology using complementarity algorithm seems to be the effective approach (Ceballos, Ehrlich, Soberón, Salazar, & Fay, 2005; Chadés et al., 2014; Chen, Zhang, Jiang, Nielsen, & He, 2016; Kullberg et al., 2015; Reyers, van Jaarsveld, & Krüger, 2000). The complementary set of the six taxa is defined as a set of sites that complement each other in terms of species composition and constitute the minimal set of sites that cover all species. Therefore, it is the focus and priority areas for biodiversity conservation and is an ideal reference frame to evaluate ecological representation. The complementarity score (*C*_*jk*_) between county *j* and county *k* was defined as follows (Colwell & Coddington, 1994):

(1) $$C_{\mathrm{jk}}=1-V_{\mathrm{jk}}/S_{\mathrm{jk}}$$

where *S*_*jk*_ **= *S*_*j*_ + *S*_*k*_ – *V*_*jk*_; *S*_*j*_ is the number of species in county *j*;* S*_*k*_ is the number of species in county *k*;* V*_*jk*_ is the number of common species both in county *j* and county *k*. The resulting *C*_*jk*_ ranges between 0 and 1.

We made an analysis of all species or threatened species via complementarity algorithm as follows:

- Select the county with the richest species for six taxa and add this county to the complementary set (*U*);
- For nationally protected species or threatened species, if a county *i* does not belong to *U*, calculate the complementarity score between county *i* and counties in *U*, select the county with the greatest complementarity score (if the greatest complementarity score is the same for several counties, select the county with the greatest species richness) and add this county to *U*, until *U* covers all species; and
- For other species except nationally protected species and threatened species, if a county *i* does not belong to *U*, calculate the complementarity score between county *i* and counties in *U*, select the county with the highest complementarity score and add this county to *U*, until all species are covered.

First, we selected the county with the greatest number of species or threatened species. All species found in this county were then excluded from further consideration. Then, we searched for the county with the greatest number of species that were not already selected (Dobson, Rodriguez, Roberts, & Wilcove, 1997). Ties for complementarity score were broken by selecting the county with the largest species richness. This process continues until all species are covered.

### 2.4 Ecological representation of PA network

Ecological representation of PA network (*I*_E_) was defined as follows:

(2) $$I_{E}=\left[a/\left(a+b\right)\right]\times 100\%$$

where the set of counties where PAs exist or PA coverage is ≥a threshold (10%, 20%, 30%, or 40%, respectively) was denoted as *S*_*P*_; *a* is the number of common counties both in the complementary set and *S*_*P*_, *b* is the number of counties in the complementary set that are not in *S*_*P*_. PA coverage was calculated as the percentage of the area of PAs in a county.

### 2.5 Effects of data errors in species distribution on CSs

The measure of species representation in PAs is often sensitive to CSs. The performance of CSs may be influenced by geographical sampling bias (omission errors and commission errors; Supporting information Appendix S1), especially by the distribution of rare species. Herein, we performed a bootstrap procedure with stratified random sampling (Muir, Wallace, Done, & Aguirre, 2015; Rizopoulos, 2009; Tille, 2015; Xu et al., 2016). To guarantee the complete coverage of environmental conditions in the study region, we employed the stratification system according to the phytogeographic regions for plants and zoogeographical regions for vertebrates in China (Wu, Sun, Zhou, Li, & Peng, 2010; Zhang, 2011). We observed two principles in this procedure: The first is that the target region (i.e. whole China) should remain unchanged, and the second is that sampling units (i.e. the basic assessment units) should be randomly selected (Xu et al., 2015, 2016). The procedure is implemented as follows: (a) Stratified random sampling was adopted to generate a sample of 60% of the total dataset from each stratum (Muir et al., 2015)_;_ (b) we created CSs based on the subset of data (60%); (c) we calculated the proportional overlap (Prendergast, Quinn, Lawton, Eversham, & Gibbons, 1993; Reyers et al., 2000) (*N*_c_/*N*_s_, where the original CS is *S*_c_, the CS based on the subset of 60% data is *S*_60%_, *N*_s_ is the number of counties in *S*_60%_, *N*_c_ is the number of common counties both in *S*_60%_ and *S*_c_) between the original CS and the CS generated based on the subset; (d) the above steps from (a) to (c) were repeated 1,000 times with randomly generated samples for most of biological taxa. Due to the great number of species, 200 replicates were carried out for woody plants to avoid very long computation time. We averaged proportional overlaps in the procedure with 1,000 replicates for ferns and vertebrates and 200 replicates for woody plants; (e) we then randomly resampled 70%, 80%, and 90% of total dataset, respectively, and repeated the above steps from (a) to (d). If proportional overlaps were relatively high, we can effectively control the impact of sampling bias on CSs and ensure the robustness of our results.

## 3 RESULTS

### 3.1 Establishment of CSs through a complementarity algorithm

**Figure 1:**
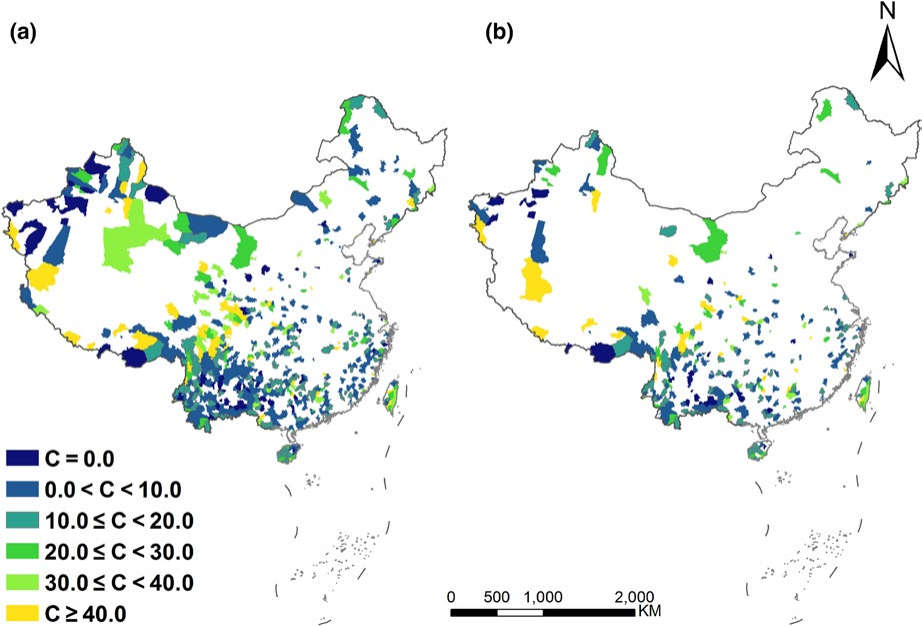
Complementary sets (CSs) for all species or threatened species of woody plants, ferns, amphibians, reptiles, birds, and mammals in the terrestrial and inland water ecosystems of China. (a) All species (*n* = 552 counties); (b) threatened species (*n* = 276 counties). C indicates protected area coverage in counties in 2013. Threatened species are those species that are listed by the IUCN Red List as Critically Endangered, Endangered, or Vulnerable. Colors other than white indicated sites (counties) of CSs

We identified CSs of counties that represent all species or threatened species of plant and vertebrate species at least once using a complementarity algorithm (Colwell & Coddington, 1994; Figure 1). We considered all species and threatened species, respectively, because their geographical patterns, importance, and conservation urgency are different (Ceballos & Ehrlich, 2006; Orme et al., 2005) and threatened species are more likely to go extinct. We evaluated the effectiveness of CSs in representing species richness. We calculated the number of all species or threatened species of woody plants, ferns, amphibians, reptiles, birds, and mammals in CSs that are located in relevant phytogeographic or zoogeographical regions (Wu et al., 2010; Zhang, 2011). These phytogeographic or zoogeographical regions were defined according to climate, topography, soil, fauna, and flora in China (Wu et al., 2010; Zhang, 2011). The number of species in CSs accounted for a large proportion of the total species number in relevant phytogeographic or zoogeographical regions (mean: 90.9%, standard deviation: 9.6 for all species of six taxa; and mean: 89.1%, standard deviation: 12.0 for threatened species of six taxa; Supporting information Table S1). It indicated that CSs represented the majority of species in each phytogeographic or zoogeographical region. Meanwhile, CSs covered all global terrestrial ecoregions (Olson & Dinerstein, 2002) (Supporting information Figure S1) and global biodiversity hotspots (Mittermeier et al., 2005) (Supporting information Figure S2) that are located in or intersected with China. Hot spots identified by other methods based on richness (Huang et al., 2012; Tang et al., 2006; Xu et al., 2016), endemism (Huang et al., 2012; Tang et al., 2006) or phylogenetic diversity (Huang et al., 2012) were mainly distributed in the broad areas between the Qinling Mountains and farther south and the eastern part of the Qinghai‐Tibetan Plateau and to the east of the plateau. In addition to the above regions, we also identified important areas in northeastern and northwestern China that were rarely considered before (Figure 1), such as the Changbai Mountains, the Da Hinggan Mountains, the Helan Mountains, the Qilian Mountains, the Tianshan Mountains, and the Altai Mountains. It means that CSs constructed in this study were geographically representative, which facilitate to confirm global conservation priorities and identify other important biodiversity areas.

### 3.2 Ecological representation in PA network

We made an assessment of 3,627 PAs which represent majority of PAs in China (Supporting information Figures S3–S5). The PA network covers a total area of 161.7 million ha, accounting for approximately 16.8% of the national terrestrial territory. Thus China’ PA network has almost met the criterion of Aichi Target 11 in terms of area percentage (17%). Most of PAs (83.1% of the total area) were distributed in nine provinces and autonomous regions in western and northeastern China, that is, Gansu, Heilongjiang, Inner Mongolia, Jilin, Liaoning, Qinghai, Sichuan, Tibet, and Xinjiang (PA coverage >12% in each province or autonomous region and even >30% in some regions), with dozens of huge PAs (>0.2 million ha each) nested within these regions. However, coverage of PAs is much lower in other provinces and autonomous regions (mostly <10%, and even <5% in some provinces) with many small PAs (Supporting information Figure S3).

**Figure 2:**
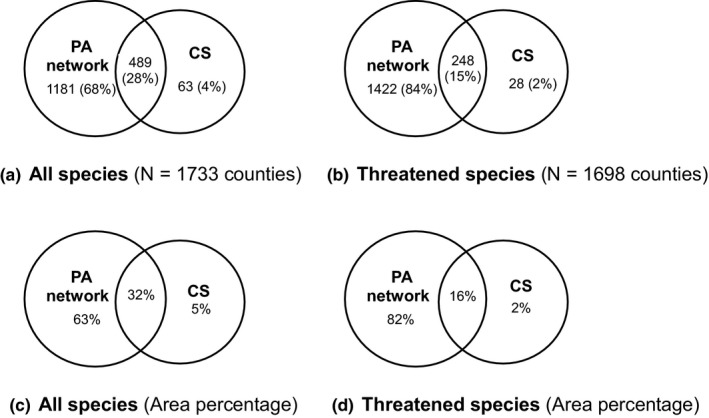
Congruence between the protected area (PA) network in 2013 and complementary sets (CSs). There were 1,670 counties where PAs exist in 2013. *N* refers to the number of counties in the union of CS and the set of the counties where PAs were present. Number refers to the number of relevant counties. Percentages in (a) and (b) are of the number of relevant counties in *N* counties. Area percentages in (c) and (d) are the proportions of the area of relevant counties in *N* counties

We compared the distribution of PAs with CSs to evaluate ecological representation. Ecological representation is defined as the percentage of the number of counties where PAs exist or PA coverage is ≥a threshold (10%, 20%, 30%, or 40%, respectively) among the total number of counties in CS. Firstly, we considered whether a PA was present in the counties of CSs regardless of PA coverage. Ecological representation has increased gradually since 1993 and exceeded 85% after 2005 (Supporting information Figure S6), which suggests that much progress has been made in China’s in situ biodiversity conservation (Ministry of Environmental Protection of China, 2014). However, there were 63 conservation gaps (counties) and 155 species (including 20 threatened species and 99 species endemic to China) that were unique for 63 gap counties were not covered by existing PAs. Furthermore, the number of counties with PAs and nested within CSs was low (489 [28%] for all species and 248 [15%] for threatened species), and the area percentages of counties with PAs and nested within CSs were low (32% for all species and 16% for threatened species; Figure 2).

**Figure 3:**
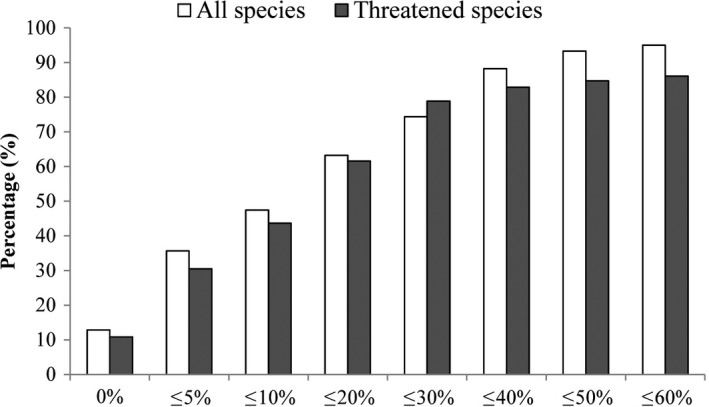
Area percentages of counties within which protected area coverage meets a threshold among the total area of complementary sets (CSs). The thresholds (*X* axis) were 0%, 5%, 10%, 20%, 30%, 40%, 50%, or 60% of the area of a county of CSs, respectively. It means large conservation gaps in counties of CSs

We further considered PA coverage in relevant counties of CSs according to different thresholds (PA coverage is ≥10%, 20%, 30%, and 40%, respectively). Different thresholds indicate the extent to which a county’s biodiversity is protected and thus reflect the level of representation. When the threshold of PA coverage changes from 10% to 40%, the numbers of counties that meet the threshold were low (decreasing from 261 to 57 for all species and from 144 to 31 for threatened species). Accordingly, the ecological representation of PAs in 2013 decreased from 47.3% to 10.3% for all species and from 52.2% to 11.2% for threatened species. Meanwhile, the area percentages of counties within which PA coverage is less than or equal to 5%, 10%, 20%, or 30% among total area of CSs are high (Figure 3). Both Figures 3 and 1 confirmed the conclusion that most spatially representative and complementary sites for biodiversity are poorly covered. We concluded that historic designation of protected areas has been inefficient in meeting conservation targets in terms of ecological representation, and a fairly large proportion of protected areas is not designed to efficiently represent biodiversity at the national scale. Although Aichi Target 11 is almost met in terms of area percentage (16.8% vs. 17%), China has a long way to go in realizing its quality target for conservation. We found that the proportional overlaps between the original CS and the CS generated based on the subsets (60%, 70%, 80%, and 90% of total dataset) were relatively high (Supporting information Table S2). Therefore, we conclude that the results in this study are robust.

## 4 DISCUSSION

Through compiling the nearly complete database of species distribution covering very broad taxonomic scope, we performed a systematic assessment of ecological representation of PA network across China. Our study revealed that low ecological representation of PA network was extensively present in China. The complementary sets are defined to cover all species, complement each other in terms of species composition, and constitute the minimal priority areas for biodiversity conservation. To reach Aichi Target 11 in terms of ecological representation, high PA coverage in CSs should be expected at the national scale. For instance, each Important Bird and Biodiversity Area (IBA) and each Alliance for Zero Extinction site (AZE) had, on average, 45% and 35% PA coverage in 2013, respectively (Juffe‐Bignoli et al., 2014). However, among 552 counties of CSs for all species in China, 63 counties had no PAs, PA coverage of 131 counties was <5% and larger than 0, PA coverage of 97 counties was <10% and larger than 5%, PA coverage of 120 counties was <20% and larger than 10%, and PA coverage of 46 counties was <30% and larger than 20% (Figure 3). In particular, there were 86 counties of CSs with PA coverage <30% in Yunnan Province, 37 in Guangxi Autonomous Region, 35 in Guizhou Province and Xinjiang Autonomous Region, respectively, 34 in Sichuan Province, 30 in Guangdong Province, 24 in Zhejiang Province, 18 Hunan Province, 16 in Hainan Province and Fujian Province, respectively, 15 in Tibet Autonomous Region, 10 in Shaanxi Province and Jiangxi Province, respectively, and 8 in Hubei Province, Inner Mongolia Autonomous Region and Chongqing City, respectively. It demonstrates that most spatially representative and complementary sites for biodiversity are poorly covered, and a fairly large proportion of protected areas is not designed to efficiently represent biodiversity at the national scale.

Similar findings were reported both at global and national levels. Globally, 17% of 4,118 threatened vertebrates were not found in a single PA and 85% were not adequately covered because PAs are biased toward locations that are cheap for conservation and away from important areas for biodiversity (Venter et al., 2014). A global assessment showed that 91% of migratory bird species have inadequate PA coverage for at least one part of their annual cycle (Runge et al., 2015). At the national scale, González‐Maya, Víquez‐R, Belant, and Ceballos (2015) found low complementarity among PAs for representing mammal species in Costa Rica and highlighted the need for greater complementarity and representativeness. Jenkins, van Houtan, Pimm, and Sexton (2015) also discovered that PAs in the United States do not adequately cover the country’s unique species, because most of its PAs are currently located in the western regions while the vulnerable species largely inhabit in the southeastern regions.

Low ecological representation of PA network may result from lack of a top‐down design and a national strategy in China. PAs were found to be generally designed in an opportunistic manner (Liu et al., 2003) rather than based on systematic conservation planning (Margules & Pressey, 2000). Most PAs were initiated and established by governments at the county and prefecture levels. Principles of systematic conservation planning might not be fully understood and implemented by local governments. PAs were designated based on intuitive understanding and partial survey. Furthermore, incentive measures for the designation of PAs were absent at the national scale. Local governments were responsible for the establishment, management, and operation of PAs, which reduce their enthusiasm to designate new PAs. Under the pressure of economic development, some PAs were even downgraded, downsized, or degazetted. Some areas important for biodiversity may not be included in PA network. Therefore, national strategies and incentive measures for PA development should be designed and implemented to promote the enlargement of existing PAs and designation of new PAs based on systematic conservation planning. Provinces and regions such as Yunnan, Guangxi, Guizhou, Xinjiang, Sichuan, Guangdong, Zhejiang, Hunan, Hainan, Fujian, Tibet, Shaanxi, and Jiangxi play an important role in enhancing ecological representation of PAs in China.

Our data are derived from county‐based presence records rather than species distribution maps, because species presence data were documented at the county level in most literatures on the distribution of vertebrates and plants and collection information of specimens in herbaria. Theoretically, richness data should be derived from survey and monitoring activities based on grids such as 1 km × 1 km or 10 km × 1 km. However, such sophisticated survey or monitoring programs are not available at the national level in China. We have to derive richness data from the literature and field surveys that were not carried out at grids but in the administrative areas, mountains, or watersheds. Furthermore, the presence records can offer more accurate information about species distribution and thus reduce commission errors to a larger extent, as presence data are collected based on actual records of species distribution in counties.

Methods of spatial prioritization for biodiversity conservation started from complementarity analysis that operated on relatively simple presence/absence datasets (Sarkar et al., 2006). The crucial concept of complementarity was that, if the goal was to represent biodiversity maximally in a region, then sites should be selected to maximize the differences in their species features (Sarkar et al., 2006). The complementarity rules have been incorporated into several planning tools including C‐Plan (Reyers, 2004) and WorldMap (Williams, 2001). More recently, methods were developed to deal with various cost factors and species‐specific connectivity and uncertainty, and conservation planning tools have become able to deal with much larger landscapes and more complicated datasets (Kukkala & Moilanen, 2012; Lehtomäki & Moilanen, 2013). Zonation developed a priority ranking of the entire landscape, in which the least useful sites received the lowest ranks and areas most valuable for biodiversity got the highest ranks (Lehtomäki & Moilanen, 2013). During the process, a visualized priority rank map and the performance curves were produced. Zonation had many analysis features, including connectivity methods, and is suited for large‐scale high‐resolution analysis (Lehtomäki & Moilanen, 2013). However, if the datasets do not meet expected requirements, the utility of Zonation may be compromised. Zonation was different from Marxan (Possingham, Ball, & Andelman, 2000), Marxan with zones (Watts et al., 2009), and ConsNet (Ciarleglio, Barnes, & Sarkar, 2009) in that it produced a priority ranking through the landscape instead of a target‐based solution. Zonation was suitable for deterministic computation on large grids, while Marxan, Marxan with zones, and ConsNet were intended to stochastic optimization on a polygon‐based description of the landscape (Lehtomäki & Moilanen, 2013). Marxan with zones was developed for allocating alternative conservation actions, while Marxan, ConsNet, and Zonation mainly deal with binary planning problems. C‐Plan was an interactive planning platform and differed from other tools that apply optimization. Meanwhile, methods to address connectivity, uncertainty, environment types, and administrative division of the landscape, etc., differed greatly between these conservation planning tools (Lehtomäki & Moilanen, 2013). If more data on costs, connectivity, and uncertainty were available in the future, besides presence/absence data, other conservation planning tools such as Zonation can be further applied in our study.

Some potential caveats to our analysis warrant consideration. First, we considered specific species rather than the species with pending taxonomic status in this study. For instance, the number of mammal species (590 species) is a bit different from that documented in the latest China’s Red List (672 (sub)species (Ministry of Environmental Protection of China and Chinese Academy of Sciences, 2015). Thereinto, 60 subspecies were merged into the parent species here. In addition, 11 new species in the latter checklist discovered through molecular techniques were still in dispute among zoologists (Jiang et al., 2015) and thus eliminated from this study. Second, we assumed that all species could be protected in counties where PAs exist or PA coverage was ≥*a* threshold. It is also assumed that all PAs could provide an equally high level of protection of biodiversity elements they contain. We did not incorporate any information on the varying levels of management effectiveness. Many nominally PAs were found to be protected only on paper, and some of PAs were poorly managed, without professional staff, clear spatial boundary, or even an administration body (Xu & Melick, 2007). These factors may reduce PAs’ effectiveness in protecting biodiversity, as the ecological representation of PAs needs further maintenance through effective and equitable management to a large extent. Thus, our analysis may overestimate the ecological representation in PAs. Finally, invertebrate species were not considered in this study. The inclusion of invertebrate species is likely to increase the area required for protection because of little congruence between complementary sets of different taxa (Orme et al., 2005; van Jaarsveld et al., 1998).

In summary, to improve the ecological representativeness of PA network across China, we recommend that multidimensional measures in terms of social, legislative, and political facets should simultaneously be improved along with the top‐down design of PA network based on systematic conservation planning. First, the promulgation of the law on PAs to upgrade the current regulations on PAs can clarify the critical roles and strategies of PAs toward sustainable development, and restructure the administrative framework for PAs (Zhang et al., 2016). Second, improved governance of PAs involved in local communities through participatory approach can enhance management effectiveness of PAs (Xu & Melick, 2007; Zhang et al., 2016). Third, mobilization of financial resources including through ecosystem service payments can provide incentives for PAs (Maiorano et al., 2015; Watson, Dudley, Segan, & Hockings, 2014; Zhang et al., 2016). Finally, regular biodiversity survey and monitoring can facilitate informed decision making at regional, national, and local levels.

## CONFLICT OF INTEREST

None declared.

## AUTHOR CONTRIBUTIONS

Haigen Xu, Mingchang Cao, Yimin Li, and Zhi Wang designed the study. Haigen Xu, Mingchang Cao, Yi Wu, Zhi Wang, Yimin Li, and Wanggu Xu analyzed data and created figures. Haigen Xu, Yun Cao, Yimin Li, and Mingchang Cao wrote the manuscript. Haigen Xu, Mingchang Cao, Zhi Wang, Yi Wu, Jun Wu, Zhifang Le, Peng Cui, Hui Ding, Wanggu Xu, Hua Peng, Jianping Jiang, Yuhu Wu, Xuelong Jiang, Zhiyun Zhang, Dingqi Rao, Jianqiang Li, Fumin Lei, Nianhe Xia, Lianxian Han, Wei Cao, Jiayu Wu, Xin Xia, and Yimin Li collected data and discussed the results.

## DATA ACCESSIBILITY

The data supporting the findings of this study are available within the article and the Supporting Information.
