# Supplementary material for: Low ecological representation in the protected area network of China
Source: Ecol Evol. 2018 May 24;8(12):6290–8. doi: 10.1002/ece3.4175 (PMC6024119; doi:10.1002/ece3.4175)
Supplement: Supplementary file 1 [file ECE3-8-6290-s001.docx]

**Low ecological representation in the protected area network of China**

Haigen Xu*^1^, Mingchang Cao^1^, Zhi Wang^1^, Yi Wu^2^, Yun Cao^1,3^, Jun Wu^1^, Zhifang Le^1^, Peng Cui^1^, Hui Ding^1^, Wanggu Xu^1^, Hua Peng^4^, Jianping Jiang^5^, Yuhu Wu^6^, Xuelong Jiang^7^, Zhiyun Zhang^8^, Dingqi Rao^7^, Jianqiang Li^9^, Fumin Lei^10^, Nianhe Xia^11^, Lianxian Han^12^, Wei Cao^13^, Jiayu Wu^14^, Xin Xia^1^, Yimin Li*^15^

*Corresponding authors. E-mail: xhg@nies.org or llym@ujs.edu.cn.

**This PDF file includes:**

Supplementary Text

Figures S1 to S6

Tables S1 to S2

References

**Appendix S1**

**Species data**

We collected the data on species distribution in relevant counties in terrestrial and inland water ecosystems of China from three different sources ^1-3^: (i) approximately 900 literature on the distribution of woody plants, ferns and vertebrates, from 1970 to 2012, e.g., *Flora Reipulicae Popularis Sinicae* ^4^, *Flora of China* ^5^, *Higher Plants of China* ^6^, *Fauna Sinica* ^7^, *A Checklist on the Classification and Distribution of the Birds of China* ^8^, *A Complete Checklist of Mammal Species and Subspecies in China* ^9^ and *China’s Mammalian Diversity* ^10^, *Colored Atlas of Chinese Amphibians* ^11^, regional and provincial monographs on floras and faunas, e.g., *Flora Yunnanica* ^12^, *Mammals of Beijing* ^13^, and numerous studies in biodiversity, e.g., the study of Cheng and Xiao ^14^; (ii) collection information of specimens in herbaria of more than 20 institutes and universities; and (iii) some recent ground observation information of such taxa based on records of field surveys by experts from more than 11 institutes of Chinese Academy of Sciences and over 14 universities. Among them, 80% of data on species distribution were from the literatures, 15% from collection information of specimens, and 5% from ground observation. The checklist of species was obtained from the Catalogue of Life China 2011 Annual Checklist ^15^ and Red Data Book of Biodiversity ^10,16-17^ (Data S1 to S6). To improve the data quality, we organized more than 20 expert meetings and invited over 100 experts specialized in different specific taxa to check the data on spatial distribution of each species across the country based on a GIS information system that we developed for species distribution at the county level ^1-3^.

**Data limitations in species distribution**

The measure of ecological representation in PAs is sensitive to CSs, but not to conservation efforts beyond CSs. CSs are critical to ecological representation evaluation of PAs. The performance of CSs may be influenced by geographical sampling bias ^18-19^, especially by the distribution of rare species, though the data limitations in the database have been reduced to the lowest extent ^1-3^. Two kinds of errors exist, i.e., omission errors and commission errors. Omission errors occur when a given species is not recorded in a county while, in fact, it is present in the county; and commission errors occur when a given species is recorded in a county when, in fact, it is not present in the county ^3^. The errors are not exclusive to the database of this study– in reality they are prevalent among most published databases of species distribution at all scales ^20-21^.

**(i) Omission errors.** Sampling efforts are not even in space ^18-20,22^. Most data on species distribution are derived from opportunistic collections rather than unified sampling strategies to reflect the full variation of environmental features in the study region ^3^. Sampling biases are common as records may be spatially biased towards more popular species or easily accessible regions ^20,23^. More omission errors may be found in the records on some poorly known species and regions ^3^. As detailed surveys across the entire possible range are rarely conducted due to lack of resources ^24,25^, a lot of species that are actually present have not yet been recorded. Omission errors may occur in species distribution data since very few counties in China have been surveyed to generate complete species lists ^26^.

**(ii) Commission errors.** Species may be misidentified or the locations may be wrongly recorded, which results in commission errors ^20,27^. Species occurrence is not stable as they move dynamically in time and space ^3^. Due to change or loss of habitats, e.g. conversion of forests to grasslands and wetlands to rice paddies, the distribution of species is likely to change. Hence, data on species distribution from the literature or specimen records may overestimate species distribution, resulting in commission errors ^3^.

**Data limitations in PAs**

**(i) Sampling errors in PAs.** There are various types of PAs in China, such as nature reserves, national parks (or scenic spots at national level), national forest parks, etc. Most types of PAs in China were considered in this study, except nature reserves of geologic relicts and paleontologic relicts, and marine nature reserves in mainland China. Nature reserves that are only depicted on paper and lack valid information on the exact geographical location were also excluded. However, the area of those excluded nature reserves was very small (less than 0.4% of the country’s land area). Hence, PAs in this study represent the majority of PAs in China. Data on PAs were obtained from official statistics, except that PAs are intersected with several counties and data on the exact area among these counties are unavailable. In such a case, we allocated the area of the PA evenly among these counties, which may lead to some errors in the calculation of ecological representation. However, such cases are very few (less than 100) and will not change the results of this study. If a PA lies in a county or PA coverage is greater than or equal to a threshold, we assumed that all species in the county should be protected. In this regard, our analysis may overestimate the ecological representation of PAs.

**(ii) Management effectiveness of PAs.** We assumed that all PAs provide an equally high level of protection of biodiversity elements they contain. We did not incorporate any information on the varying levels of management effectiveness ^28^. It is evident that many nominally PAs were protected only on paper. Many PAs were poorly managed, without professional staff, clear spatial boundary or even an administration body. These issues were [exacerbate](http://www.baidu.com/link?url=sD5mvNH1ZN1wx3igunapJ70c3l3v6e0JPm5E8cWxeBlpX3xTFS1B7B1mDOg0K89ZhtN5-L-W5hbPVJln1yLSGGzTtEIKB4OzaJcoHY5mTYK&wd=&eqid=cf497f5b001d45f80000000556beced5)d by poor working conditions, conflicts between the objectives of generating revenue and protection, lack of funding, low salaries, and lack of community involvement and trust ^29^. Rising management effectiveness of PAs can also improve ecological representation. However, data on management effectiveness is unavailable in this study. Thus, our results only based on coverage may overestimate protection in reality.


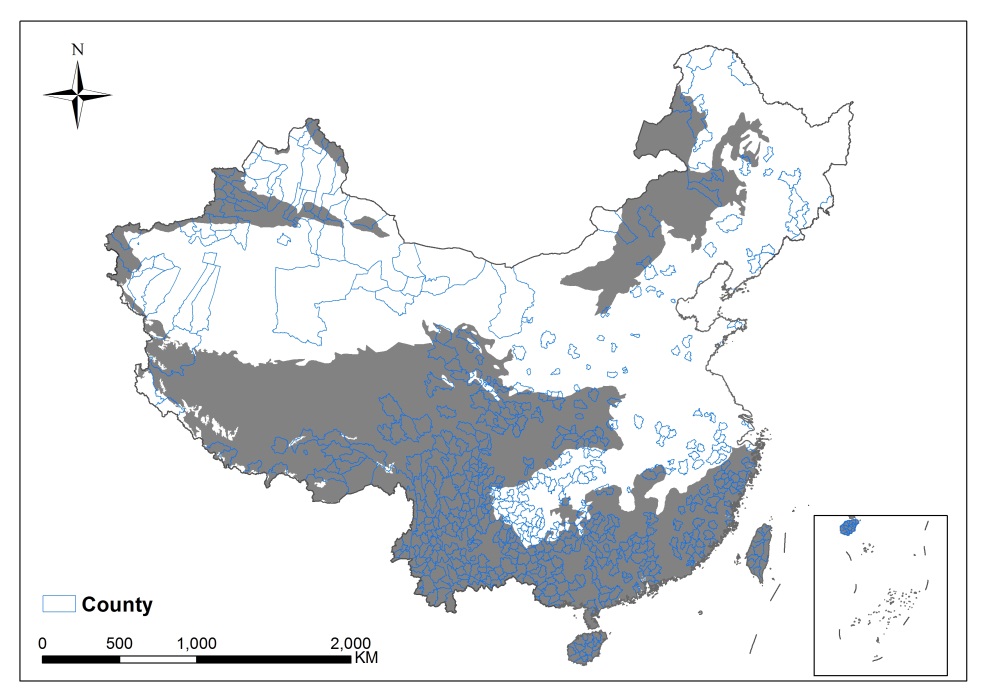


**Figure S1 CS for all species covers terrestrial global ecoregions (grey) ^30^ that are located in or intersected with China.** Counties in CS for all species were described in blue color.


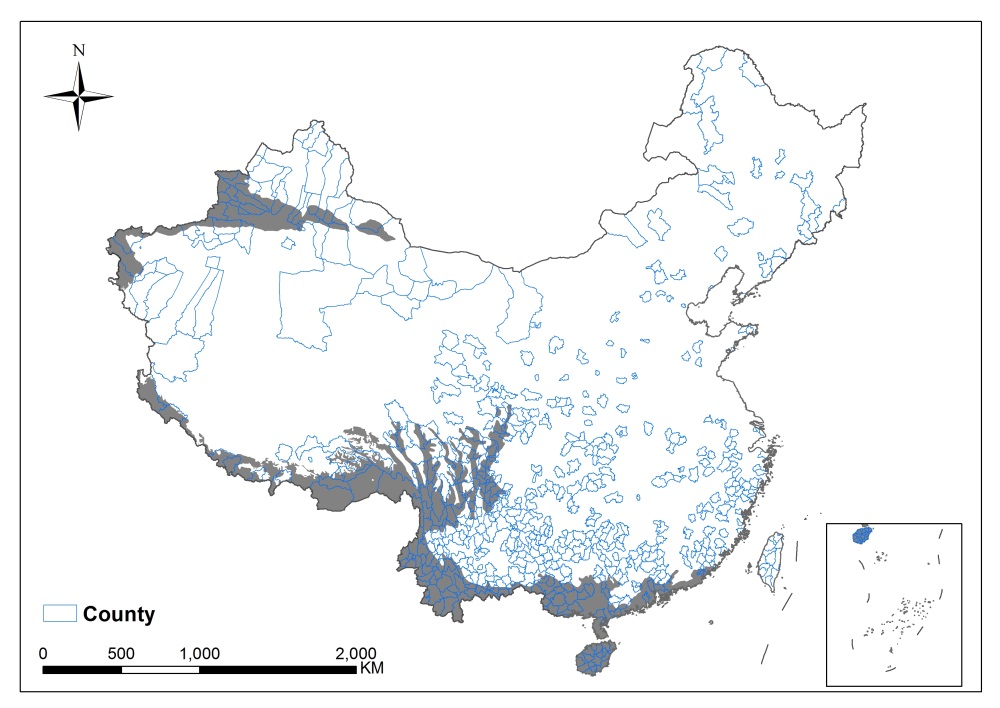


**Figure S2 CS for all species covers four global biodiversity hotspots (grey) ^31^ that are located in or intersected with China.** Counties in CS for all species were described in blue color.


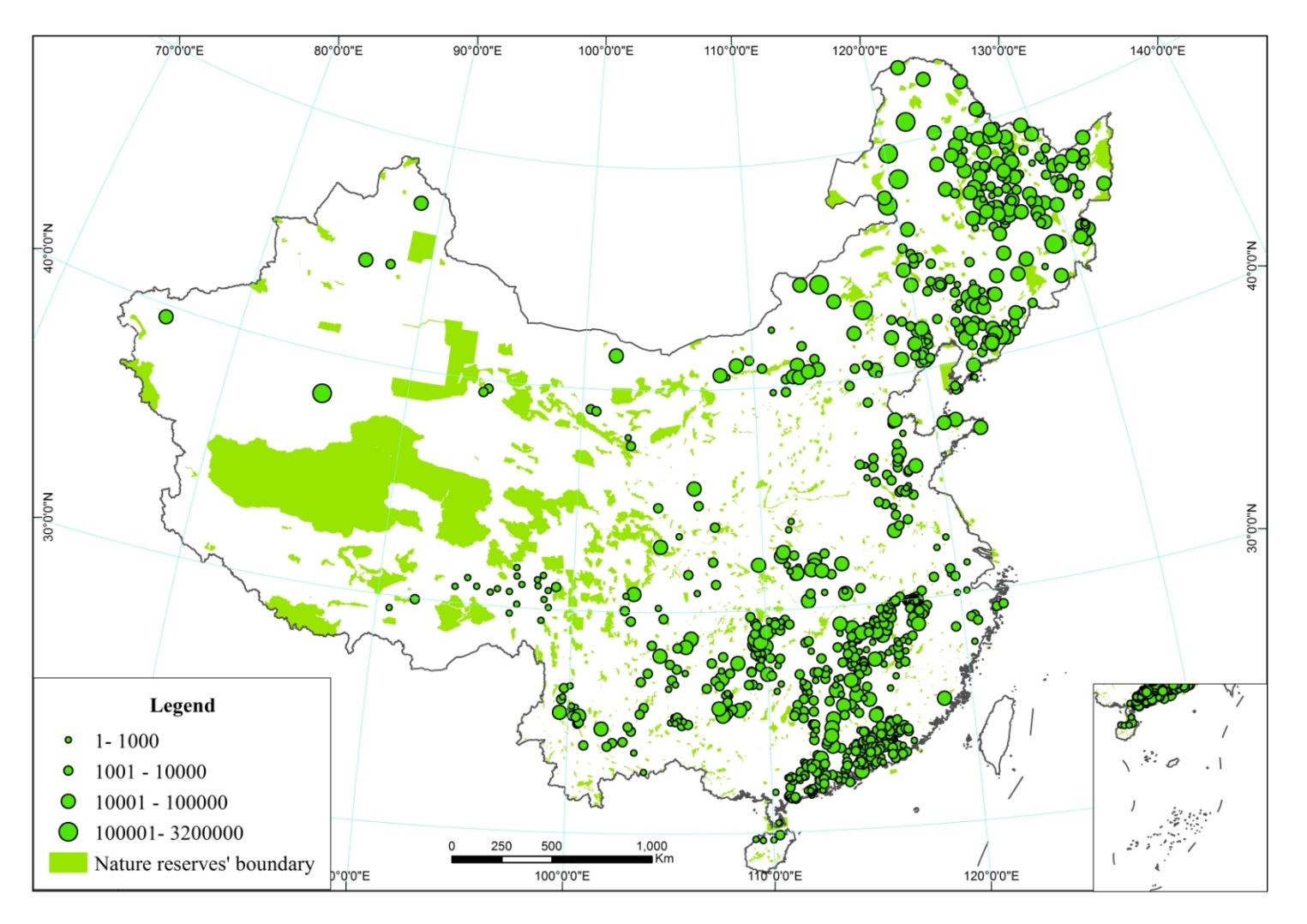


**Figure S3 Spatial distribution of nature reserves in Mainland China in 2013**. Nature reserves of geologic relicts and paleontologic relicts and marine nature reserves were excluded from this study. PAs that are only depicted on paper and lack valid information on exact geographical location were also eliminated. Nature reserves with valid boundary were described in green color, otherwise by dots. The size of dots is described in the legend (unit: ha).


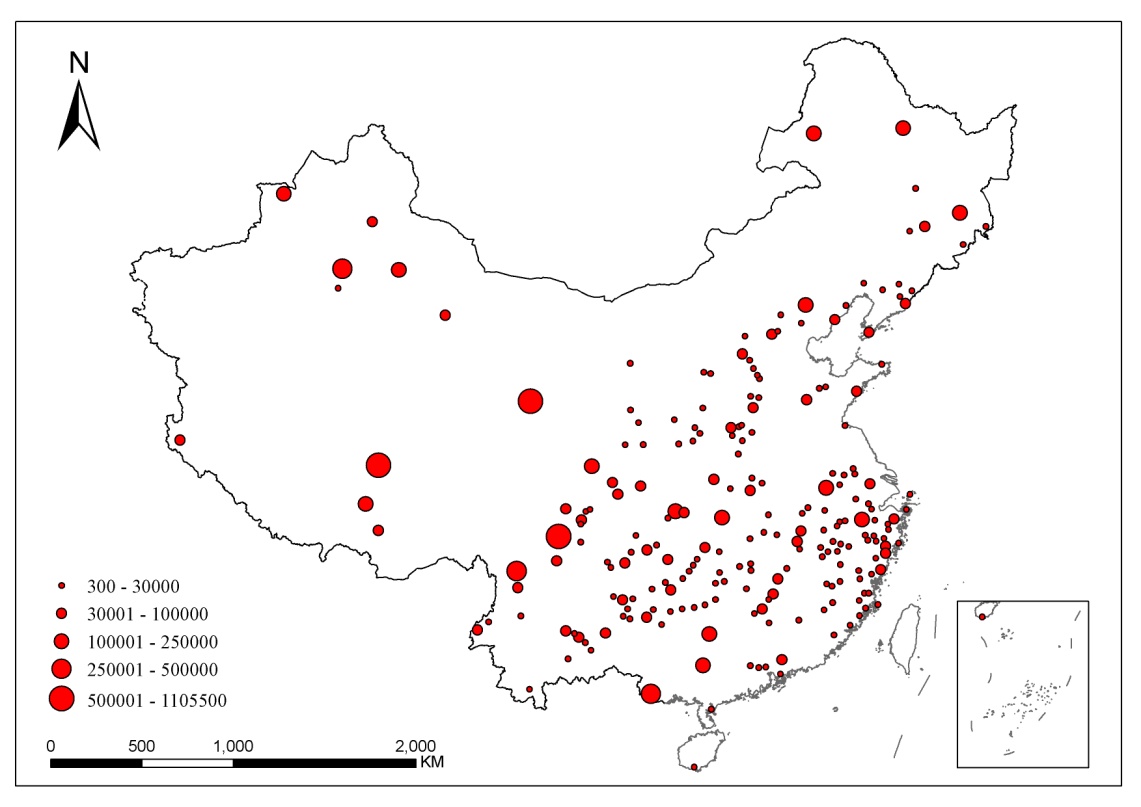


**Figure S4 Spatial distribution of 225 national parks in Mainland China in 2013.** The size of dots is described in the legend (unit: ha).


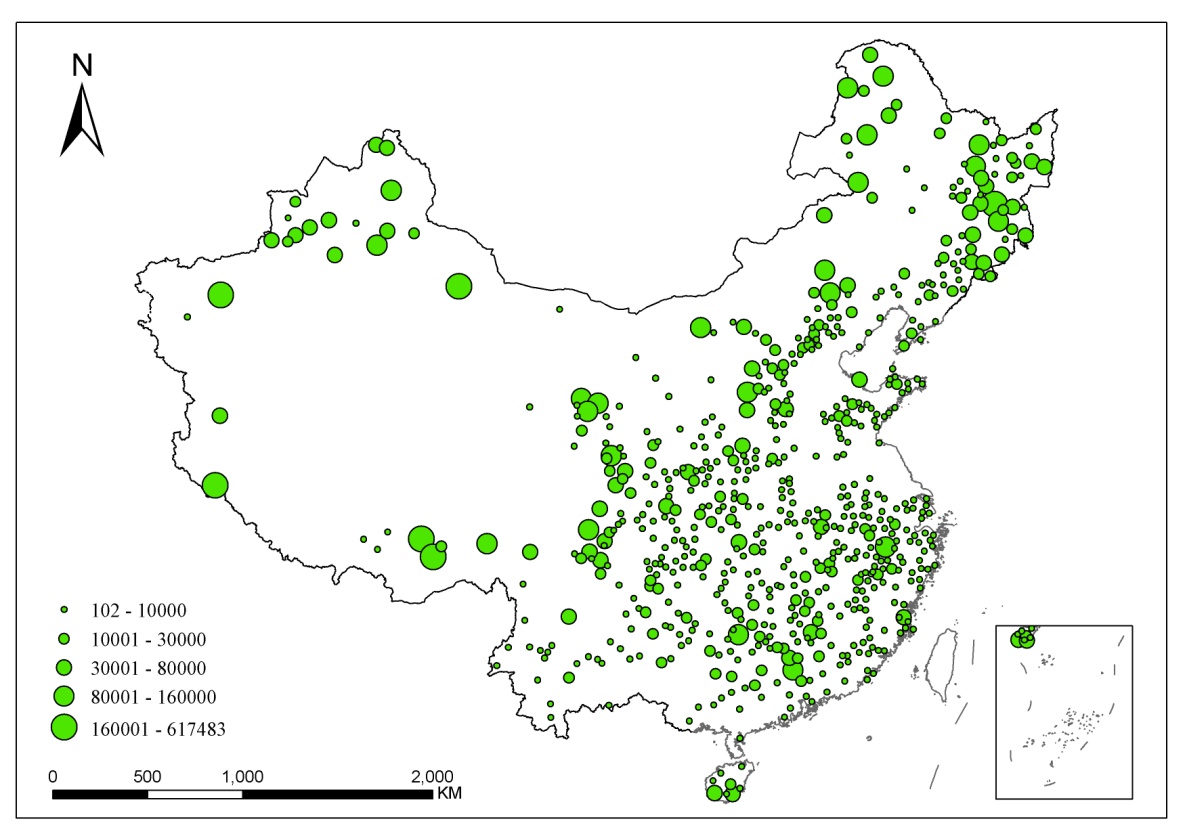


**Figure S5 Spatial distribution of 779 national forest parks in Mainland China in 2013.** The size of dots is described in the legend (unit: ha).


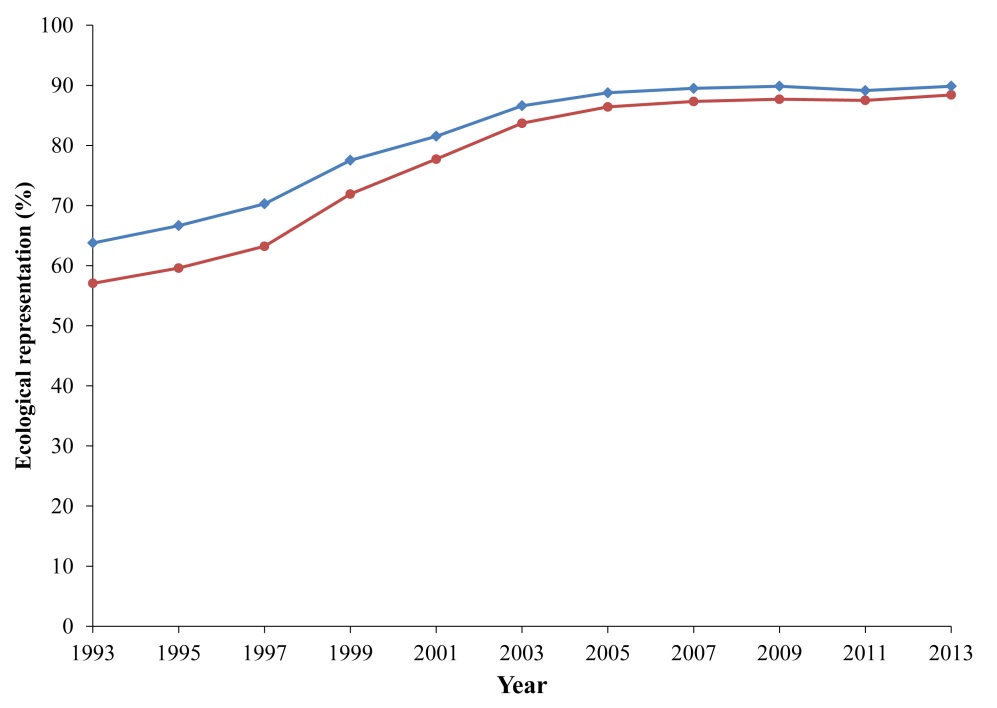


**Figure S6 Temporal change of ecological representation in PAs between 1993 and 2013.** We considered whether a PA was present in the counties of CS rather than PA coverage. Red line is for all species, and blue line for threatened species.

**Table S1** **Proportion (%) of the number of species in CSs to the total number of species in the relevant phytogeographic or zoogeographical regions.** The number of all species or threatened species of woody plants, ferns, amphibians, reptiles, birds and mammals in CSs that are located in relevant phytogeographic or zoogeographical regions ^32-33^ were calculated. Percentage in the table refers to the proportion of the number of species in CSs to the total number of species in the relevant phytogeographic or zoogeographical regions.

| **Zoogeographical/ phytogeographic regions** | **Mammals** | | **Birds** | | **Reptiles** | | **Amphibians** | | **Woody plants** | | **Ferns** | |
| --- | --- | --- | --- | --- | --- | --- | --- | --- | --- | --- | --- | --- |
|  | (a) | (b) | (a) | (b) | (a) | (b) | (a) | (b) | (a) | (b) | (a) | (b) |
| **Zoogeographical regions** |  |  |  |  |  |  |  |  |  |  |  |  |
| Northeast region | 89.4 | 92.5 | 95.4 | 89.3 | 81.9 | 69.8 | 82.1 | 99.8 |  |  |  |  |
| Central China region | 97.6 | 98.5 | 94.5 | 96.3 | 97.0 | 96.7 | 97.9 | 97.7 |  |  |  |  |
| North China region | 75 | 60.4 | 92.1 | 77.3 | 75.6 | 86.5 | 71.5 | 66.6 |  |  |  |  |
| South China region | 99.1 | 96.4 | 99.2 | 99.4 | 96.8 | 97.9 | 96.8 | 97.9 |  |  |  |  |
| Mongolia-Xinjiang region | 93.2 | 99.6 | 94.3 | 76.2 | 91.7 | 99.6 | 88.4 | 99.7 |  |  |  |  |
| Southwest region | 99.2 | 99.8 | 99.1 | 92.3 | 98.9 | 96.8 | 99.1 | 99.6 |  |  |  |  |
| Qinghai-Tibet region | 90.7 | 89.1 | 88.3 | 79.2 | 85.5 | 79.6 | 95.8 | 87.1 |  |  |  |  |
| **Phytogeographic regions** |  |  |  |  |  |  |  |  |  |  |  |  |
| Eurasian forest subkingdom |  |  |  |  |  |  |  |  | 86.2 | 88.1 | 88.1 | 66.6 |
| Eurasian steppe subkingdom |  |  |  |  |  |  |  |  | 61.9 | 60.3 | 65.2 | 66.5 |
| Sino-Japanese forest subkingdom |  |  |  |  |  |  |  |  | 96.9 | 93.8 | 98.7 | 92.9 |
| Tibetan Plateau subkingdom |  |  |  |  |  |  |  |  | 81.7 | 85.8 | 97.4 | 99.7 |
| Central Asian desert subkingdom |  |  |  |  |  |  |  |  | 88.7 | 84.9 | 91.2 | 99.6 |
| Sino-Himalayan forest subkingdom |  |  |  |  |  |  |  |  | 98.6 | 95.7 | 98.7 | 89.5 |
| Tropical Southeast Asia subkingdom |  |  |  |  |  |  |  |  | 98.9 | 98.8 | 99.3 | 98.8 |

Note: (a): all species; (b): threatened species.

**Table S2 Mean and standard deviation of the proportional overlaps between the original CS and CSs generated based on the subsets of samples (60%, 70%, 80% and 90%) from whole China**. Stratified random sampling with a bootstrap procedure was performed. We averaged overlaps in the procedure with 1,000 replicates for most of biological taxa. Due to the great number of species, 200 replicates were carried out for woody plants to avoid very long computation time. The overlaps were relatively high.

| **Taxa** | **Subsets of 60% sample** | **Subsets of 70% sample** | **Subsets of 80% sample** | **Subsets of 90% sample** |
| --- | --- | --- | --- | --- |
| Ferns | 0.696±0.041 | 0.757±0.039 | 0.822±0.033 | 0.883±0.026 |
| Woody plants | 0.750±0.024 | 0.813±0.022 | 0.876±0.017 | 0.938±0.014 |
| Amphibians | 0.577±0.065 | 0.662±0.065 | 0.745±0.061 | 0.842±0.055 |
| Reptiles | 0.539±0.070 | 0.616±0.073 | 0.708±0.071 | 0.814±0.066 |
| Birds | 0.525±0.068 | 0.608±0.071 | 0.694±0.072 | 0.805±0.068 |
| Mammals | 0.513±0.064 | 0.596±0.065 | 0.684±0.069 | 0.805±0.071 |

**References**

1. Xu HG et al. (2015) Determinants of mammal and bird species richness in China based on habitat groups. *PLoS ONE* **10**, e0143996.
2. Xu HG, Cao MC, Wu J, Ding H (2013) *Assessment Report on Biodiversity Baseline in China*. (Science Press, Beijing).
3. Xu HG et al. (2016) [Disentangling](http://www.baidu.com/link?url=tqzDlR_BaITbFCftrb9MlgEdGvYKHN2lhywAbSSnXYYmn4vmFh0otDLISgiQnytdTMDEGO7Vq2J_qwRUtcQ1Z8QKVjsrrI898TwafMBHKl_) the determinants of species richness of vascular plants and mammals from national to regional scales. *Sci. Rep.***6***, 21988*.
4. Editorial Committee of Flora Reipublicae Popularis Sinicae (1959-2004) *Flora Reipulicae Popularis Sinicae (Vol. 1-80)* (Science Press, Beijing).
5. Wu ZY, Raven PH, Hong DY (1994-2006) *Flora of China* (Missouri Botanical Garden Press, St. Louis). http://flora.huh.harvard.edu/china/mss/treatments.htm (Date of access: from 21/05/2009 to 3/11/2012).
6. Fu LG et al. (1999-2004) *Higher Plants of China (Vol. 2-13)* (Qingdao Publishing House, Qingdao).
7. Editorial Committee of Fauna Sinicae (1978-2012) *Fauna Sinicae* (Science Press, Beijing).
8. Zheng GM (2011) *A Checklist on the Classification and Distribution of the Birds of China* (Science Press, Beijing).
9. Wang YX (2003) *A Complete Checklist of Mammal Species and Subspecies in China: a Taxonomic and Geographic Reference* (China Forestry Publishing House, Beijing).
10. Jiang ZG et al. (2015) China’s mammalian diversity. *Biodiversity Science* **23**, 351-364.
11. Fei L, Ye CY, Jiang JP (2010) *Colored Atlas of Chinese Amphibians* (Sichuan Science and Technology Press, Chengdu)
12. Editorial Committee of Flora Yunnanica (1977-2006) *Flora Yunnanica (Vol. 1-21)* (Science Press, Beijing).
13. Chen W, Gao W, Fu BQ (2002) *Mammals of Beijing* (Beijing Press, Beijing).
14. Cheng RM, Xiao WF (2005) Biodiversity of main coniferous forests at low elevation of Three Gorges Reservoir Area. *Chinese Journal of Applied Ecology* **16**, 1791-1794.
15. The Biodiversity Committee of Chinese Academy of Sciences (2011) *The Catalogues of Life China 2011 Annual Checklist* (Science Press, Beijing).
16. Ministry of Environmental Protection of China and Chinese Academy of Sciences (2015) *Red List Book of Biodiversity: Vertebrates* (Available at: http://www.mep.gov.cn) (Date of access: June 30, 2015).
17. Ministry of Environmental Protection of China and Chinese Academy of Sciences (2013) *Red List Book of Biodiversity: Higher plants* (Available at: http://www.mep.gov.cn) (Date of access: August 30, 2013).
18. Hortal J, Lobo JM, Jim_enez-Valverde A (2007) Limitations of biodiversity databases: case study on seed-plant diversity in Tenerife, Canary Islands. *Conserv. Biol.* **21**, 853-863.
19. Soberón J, Jim_enez R, Golubov J, Koleff P (2007) Assessing completeness of biodiversity databases at different spatial scales. *Ecography* **30**, 152-160.
20. Boitani L et al. (2011) What spatial data do we need to develop global mammal conservation strategies? *Phil. Trans. R. Soc. B* **366**, 2623-2632.
21. Rodrigues AS et al. (2004) Effectiveness of the global PA network in representing species diversity. *Nature* **428**, 640-643.
22. Beck J, Schwanghart W (2010) Comparing measures of species diversity from incomplete inventories: an update. *Methods Eco. Evol.* **1**, 38-44.
23. Boakes EH, McGowan PJK, Fuller RA, Ding CQ, Clark NE, O’Connor K, Mace GM (2010) Distorted views of biodiversity: spatial and temporal bias in species occurrence data. *PLoS Biol.* **8**, e1000385.
24. Haila Y, Margules CR (1996) Survey research in conservation biology. *Ecography* **19**, 323-331.
25. Pressey RL (2004) Conservation planning and biodiversity: assembling the best data for the job. *Conserv. Biol.* **18**, 1677-1681.
26. Qian H and Ricklefs RE (2011) Latitude, tree species diversity and the metabolic theory of ecology. *Global Ecol. Biogeogr.* **20**, 362-365.
27. Rondinini C, Wilson KA, Boitani L, Grantham H, Possingham HP (2006) Tradeoffs of different types of species occurrence data for use in systematic conservation planning. *Ecol. Lett.* **9**, 1136-1145.
28. Ferrier S et al. (2004) Mapping more of terrestrial biodiversity for global conservation assessment. *BioScience* **54**, 1101-1109.
29. Xu JC, Melick DR (2007) Rethinking the effectiveness of public PAs in southwestern China. *Conserv. Biol.* **21**, 318–328.
30. Olson D M, Dinerstein E (2002) The global 200: priority ecoregions for global conservation. *Ann. Missouri Bot. Gard.* **89**, 199-224.
31. Mittermeier RA, Gil PR, Hoffman M, Pilgrim J, Brooks T, Mittermeier CG, Lamoreux J, Fonseca GAB (2005) *Hotspots Revisited* (The University of Chicago Press, Chicago).
32. Wu ZY, Sun H, Zhou ZK, Li DZ, Peng H (2010) Floristics of Seed Plants from China (Science Press, Beijing).
33. Zhang R Z (2011) *Zoogeography of China* (Science Press, Beijing).
